# Path planning in three-dimensional space based on butterfly optimization algorithm

**DOI:** 10.1038/s41598-024-52750-9

**Published:** 2024-01-28

**Authors:** Hakimeh Mazaheri, Salman Goli, Ali Nourollah

**Affiliations:** 1 Computer Department, Faculty of Electrical and Computer Engineering, University of Kashan, Kashan, Iran; 2 Computer Department, Faculty of Computer Engineering, Shahid Rajaee Teacher Training University, Tehran, Iran

**Keywords:** Engineering, Aerospace engineering

## Abstract

Path planning is one of the most critical issues in many related fields including UAVs. Many researchers have addressed this problem according to different conditions and limitations, but modelling the 3-D space and routing with an evolutional algorithm in such spaces is an open issue. So, in this paper, we first, introduce a method to grids the environment using geometrical shapes. This can reduce the random states of cell decomposition and increases the computational speed. We then propose an effective routing algorithm based on the butterfly optimization algorithm (BOA). It can simultaneously optimize multiple path planning objectives. It uses an objective function to compute the shortest path, based on obstacle avoidance and the UAV’s operational power minimization. A novel concept, the intelligent throwing agent, used in this algorithm prevents getting stuck in local optima and increases the network coverage in path planning. The throwing agent prevents the collision of the UAV with the obstacles using geometrical techniques and contour lines. The simulation results show that BOA has the least and second-least cost in best-case and worst-case scenarios in comparison with ant colony and particle swarm. Its run time and the optimal value of the fitting function are also better than the two other algorithms.

## Introduction

The rapid development of low-cost radio communications, global position systems, and micro-computers has given Unmanned Aerial Vehicles (UAVs) massive potential in various applications such as assist and rescue^1^, surveillance and reconnaissance^2^, patrolling the border^3^, tracking^4^, fire management^5^, traffic accidents monitoring and management^6^, goods delivery^7^, and telecommunication amplifiers^8^. These applications facilitate human life and make it more secure. UAVs have attracted the attention of many researchers and investors due to their agility, cost-effectiveness, and high maneuverability^9^. The many UAV applications come with some important challenges. These challenges differ in significance and sensitivity according to the application and network type.

### Modeling the environment

To model the environment, targets, and obstacles are modeled as geometrical shapes and stored in a data structure, e.g., a graph, whose nodes and edges are represented by points and lines^10^.

### Energy consumption

UAVs have limited energy storage. The stored energy might be just enough to fly for as little as 30 min. Vehicular ad hoc networks, on the contrary, use car batteries that are charged while the car is moving^11^.

### Mobility

Energy consumption differs with the application. UAVs in earthquake regions float over the area. Their connection is dynamic and low-speed. However, they move through large areas with a higher speed in agriculture or forest monitoring applications.

*Autonomy and collision avoidance* are challenging problems due to the dynamic topology, non-homogenous distribution, and passing through different geographical situations and crowded paths^12^. UAVs cannot solve this problem on their own^13^. To carry on various operations, the UAV should be autonomous, i.e., solve the problem and find the solution by itself^14^.

*Path planning* is a UAV utilization challenge due to the high rate of changes in UAV networks. Rapid transport and non-homogenous distribution of the UAVs increase the chance of collision and crowding of the UAVs along the path. This makes finding an optimal path from the source to the destination without obstacle collisions even more significant^13^. Path planning is proposed as an effective solution to determine targets, appropriate flight altitudes, and a path for UAVs^13^. Path planning is either covered or non-covered. Non-covered approaches search a small piece of the environment while covered approaches search a large part or all of the environment. Many path-planning algorithms, such as probabilistic, evolutional, potential fields, cell decomposition, and graph-based algorithms, have been used for that purpose^14–16^.

This research aims to introduce a path planning optimization algorithm to improve the intended parameters such as environment coverage, near-optimal path generation, avoiding collision, and reducing energy consumption. The innovations of the proposed methods are:

- Modeling the environment based on covered path planning and convex griding to reduce the random states
- Generating points between the source and destination to go around the obstacles and reduce sudden turns and intense maneuvers.
- Path planning using a meta-heuristic algorithm capable of optimizing issues such as path length, collision avoidance, and reducing energy consumption.

In Section “Problem statement”, we state the problem as steps of path planning. Section “Modeling the environment” reviews the related researches in this area and categorizes them according to the solution type and application. Section “Background” introduces BOA and our proposed algorithm to discretize the environment and plan paths. In Section “The proposed method”, the results of experiments and comparisons with other existing algorithms are presented. Section “Designing and evaluating the path in 2D space” concludes the paper and gives suggestions for further work.

## Problem statement

The problem space is defined as follows in path planning:

### Definition 1

Workspace ${\text{W}}$ is the physical space shown as ${\mathbb{R}}^{2}$ in a planar (two-dimensional) space and as ${\mathbb{R}}^{3}$ in a three-dimensional space.

### Definition 2

An obstacle ${{\text{O}}}_{{\text{i}}}$ is part of workspace ${\text{W}}$ that is permanently occupied

1 $${\{{\text{O}}}_{{\text{i}}}\in {\text{W}}|{\text{i}}=1,\dots ,{\text{n}}\}$$

### Definition 3

UAV consists of one or more solid bodies with motion limitations. It is shown as ${\text{U}}$ which shows the area occupied by the UAV body. ${\text{U}}\in {\text{W}}$.

### Definition 4

UAV configuration, ${\text{U}}({{\text{q}}}_{{\text{s}}})$, is a set of parameters such as velocity, attitude angle, and altitude which completely specifies the UAV ${\text{U}}$ position in a specific time and position ${\text{q}}$. S is a function of the time and place of the UAV. ${\text{U}}({{\text{q}}}_{{\text{s}}})$ Is a region in ${\text{W}}$ occupied by UAV ${\text{U}}$ with configuration ${\text{q}}$ in a specific moment and position s. Different combinations of parameters ${\text{represe}}$nt various degrees of freedom. The workspace is divided into three regions occupied by UAV, the obstacle, and the free configuration space.

### Definition 5

Configuration space ${\text{C}}$ for the UAV ${\text{U}}$ is the set of all configurations of ${\text{U}}$ in ${\text{W}}$

2 $${\text{C}}=\left\{\forall {{\text{q}}}_{{\text{s}}}|{\text{U}}({{\text{q}}}_{{\text{s}}})\subseteq {\text{W}}\right\}$$

### Definition 6

Obstacles of configuration space ${\text{CO}}$, is a map of the obstacles in the workspace into the configuration space. It consists of all configurations where the UAV collides with the obstacles

3 $${\text{CO}}=\{\mathrm{all }{{\text{q}}}_{{\text{s}}},{{\text{O}}}_{{\text{i}}}|{\text{U}}({{\text{q}}}_{{\text{s}}})\cap {{\text{O}}}_{{\text{i}}}\ne \mathrm{\varnothing }\}$$

### Definition 7

Free configuration space ${{\text{C}}}_{{\text{free}}}$ is the set of all configurations where the UAV does not collide with the obstacles of the workspace.

4 $${C}_{free}=\{all {q}_{s},{O}_{i}|U({q}_{s})\cap {O}_{i}=\mathrm{\varnothing }\}$$

### Definition 8

Local search begins from a solution in front of the UAV and successively moves to adjacent solutions. It’s only possible when neighboring and adjacency relations are defined in the problem’s search space. The local search leads to a local path. A local path is a continuous function shown by ${lp}_{i}({q}_{s},{q}_{s}^{\mathrm{^{\prime}}})$ in Eq. (5). ${lp}_{i}$ is the $i$th local path, ${q}_{s}$ is the UAV configuration at the beginning of the local path, ${q}_{s}^{\mathrm{^{\prime}}}$ is the UAV configuration at the destination point, and $n$ is the number of points in the local path. Showing local path as ${lp}_{i}\left({q}_{s},{q}_{s}^{\mathrm{^{\prime}}}\right)$ we have

5 $${lp}_{i}\left({q}_{s},{q}_{s}^{\mathrm{^{\prime}}}\right)=\sum_{i=1}^{n}lp\left(i , i+1\right) ,1\le i\le n ,\left[{q}_{s},{q}_{s}^{\mathrm{^{\prime}}}\right]\subseteq C$$

### Definition 9

Global path is defined similarly to the local path. The only difference is the UAV configuration at the beginning and the destination point are shown as $Gp({q}_{start},{q}_{end})$. As we use a discrete search, $Gp$ is defined as an ordered set of $k$ local paths $Gp({lp}_{1},\dots ,{lp}_{k})$. This gives a continuous path where the $k$ parameter is the number of intermediate points and ${Gp}_{k}$ is a global path with $k$ intermediate points.

6 $${Gp}_{k}\left({q}_{start},{q}_{end}\right)=\sum_{i=1}^{k}lp\left(i , i+1\right) ,1\le i\le k$$

Path planning is performed in two steps: preprocessing based on the area covered and the position of the obstacle and path search to choose the best path among the possible ones. Let's discuss these in more detail.

### Ethical approval

This material is the authors’ own original work, which has not been previously published elsewhere. The paper is not currently being considered for publication elsewhere. The paper reflects the authors’ own research and analysis in a truthful and complete manner.

## Modeling the environment

### Designing obstacles

**Figure 1:**
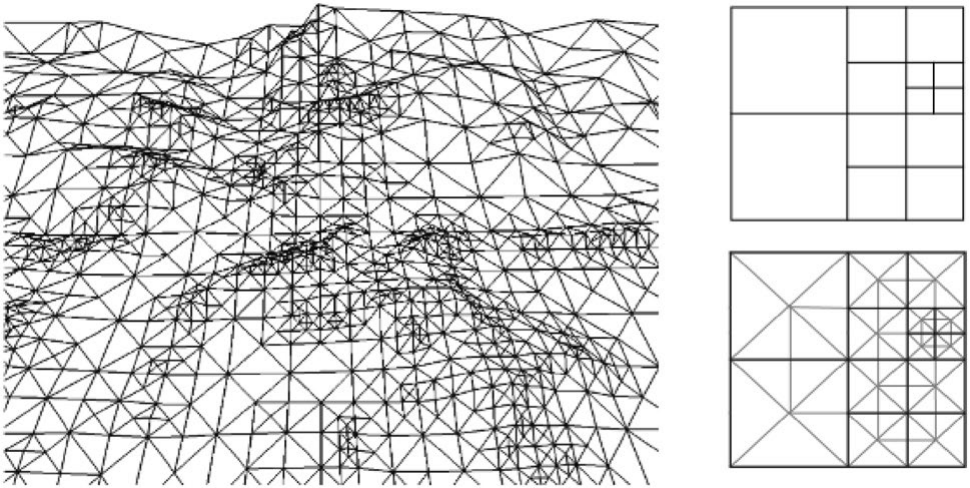
Multi-resolution terrain image discretization ^18^.

The first step in path planning is modeling the environment. It consists of discretizing the environment and designing obstacles and targets. Two-dimensional path planning methods can’t find the obstacles in 3-D environments. This needs special 3-D algorithms that consider the uncertainties of the natural world. Such algorithms require a discretization of that world^17^. Hierarchical data structures are used in most discretizing and presenting digital input data^18^. Discretization is used in applications such as visualizing multi-attitude terrain data in a multi-resolution model. These structures need to be compatible with the terrain features to reduce the complexity without affecting the image’s clarity. It should be possible to extract variable resolution data from these structures to support multi-level details in run time. Figure 1 shows Multi-resolution terrain image discretization.

**Figure 2:**
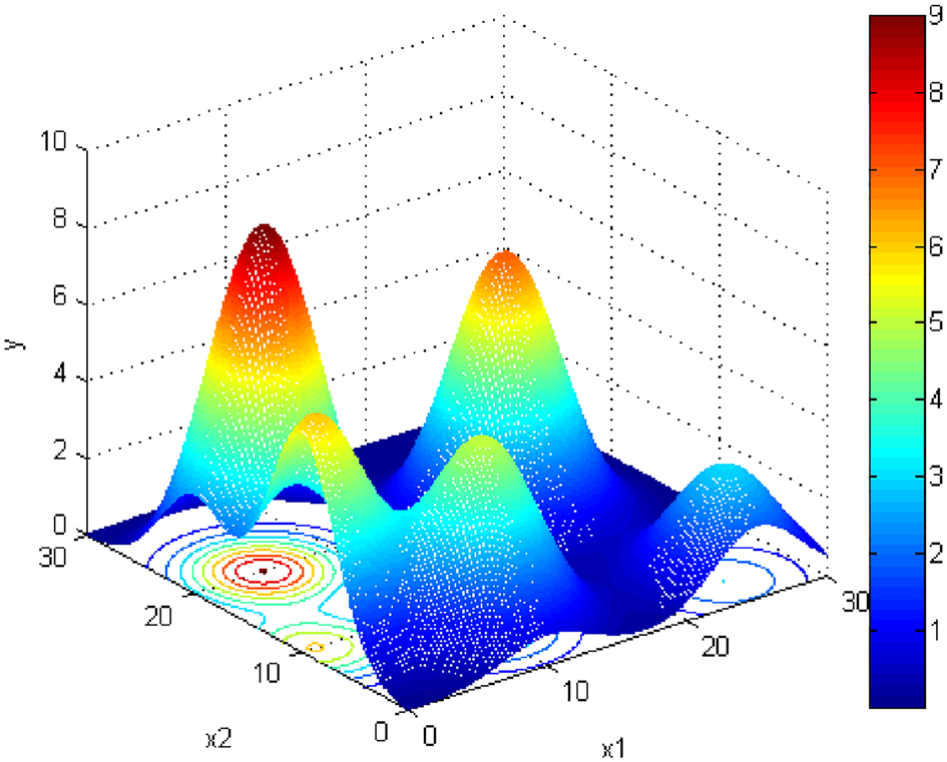
Topographic models.

UAV missions are designed based on complex three-dimensional topography. Topography is a map showing terrain features. It's crucial in designing complex environments. To simulate obstacles in complex topographies we use contour lines. These non-intersecting lines connect all terrain points of the same altitude. The smallest closed contour in such maps shows the highest or lowest point. Contour lines specify the general situation of the obstacle in topographic maps. We use a normal distribution to simulate cone-shaped obstacles. The topography used is based on the model proposed in^19^. In Eq. (7), ${h}_{i}$ is the highest altitude of the mountain, and ${a}_{i}$ and ${b}_{i}$ are the mountain’s central position. ${z}_{i}$ Is the normally distributed topographic model of the $i$th obstacle and $z$ is the normally distributed topographic model. The default value for standard deviation is 20. Figure 2 shows the topographic models including contour lines.

To compute whether we hit the cone-shaped obstacles, and if so, what would be the impact, the $x, y$, and $z$ coordinates of some line segments’ points are obtained using the three-dimensional line equation for each $x$ and $y.$ the corresponding $z$ value will be calculated as follows

7 $$\begin{gathered} z_{i} \left( {x,y} \right) = h_{i} \times e^{{\frac{{\left( {x - a_{i} } \right)^{2} }}{20}{ } - { }\frac{{\left( {x - b_{i} } \right)^{2} }}{20}}} \hfill \\ z\left( {x,y} \right) = \mathop \sum \limits_{i = 1}^{\mu } h_{i} \times e^{{\frac{{\left( {x - a_{i} } \right)^{2} }}{20} - \frac{{\left( {x - b_{i} } \right)^{2} }}{20}}} \hfill \\ \end{gathered}$$

If the $z$ value of any point is less than or equal to the $z$ obtained above, a collision happens. If none of the points on the line segment collides with the cone-shaped obstacle, the path is safe.

8 $$\frac{x-{x}_{1}}{a}=\frac{y-{y}_{1}}{b}=\frac{z-{z}_{1}}{c}=t$$

In Eq. (8) $a. b, and c$ parameters show the directions of the coordinate systems axes in the three-dimensional space. Not hitting the obstacles and going through the shortest path affect path planning. This paper proposes a meta-heuristic algorithm to solve combinational multi-target problems.

### Searching for the path

UAV limitations such as colliding with obstacles and each other, limited maneuverability, carrying a load, and environmental conditions make the design and implementation of path planning algorithms difficult. The extensive growth of UAV applications in different areas might render previous algorithms ineffective or inappropriate. In a complex environment where UAVs are surrounded and sought by complex objects, we need three-dimensional algorithms. Path planning techniques are either sampling-based or artificial intelligence based. The first group is used in preprocessing and environment design using cell decomposition^20^, roadmap^21^, or potential filed^22^ approaches. The second group is used during the search, e.g., using nature-based or otherwise meta-heuristic approaches. Genetic algorithms, evolutional models, simulated annealing, and ant colony optimizations are examples of this group^15^. Search step algorithms in this paper have better performances than the traditional ones. The rest of the paper discusses practical path-planning algorithms.

## Background

Representing the UAV path in the three-dimensional environment is the first step in path planning. Two major representation techniques, namely sampling-based and artificial intelligence based are discussed. Sampling-based methods need predefined information to configure the workspace in three-dimensional spaces. They are used in preprocessing and environment modeling. The environment is divided into many nodes distributed in the workspace using optimized path-planning algorithms. These will be used during the search.

### Preprocessing and environment modeling algorithms

The intended environment, source and destination points, obstacles positions, and other effective parameters in path-planning are determined. Cell decomposition is a sample-based practical approach for this purpose. Many researchers have studied cell decomposition for preprocessing and environment modeling. These have been categorized according to decomposing or not decomposing the cell.

#### Decomposing and discretizing

In decomposing approaches, the environment is usually divided into non-intersection regions called cells. The size and resolution of the cells vary according to the decomposition type. This approach ensures that each cell is visited exactly once and the whole environment is covered. Larger cells might need several moves to cover, while one move might be enough in smaller cells. These cells are usually single-robot size (for terrain covering) or the size of the range of the sensor or camera (for aerial coverage). Covered path planning includes the target region, cell decomposition methods, performance criteria, and availability of information. Cooperative, round-trip, decentralized, and line-forming path planning are examples of decomposition-based algorithms^23,24^.

#### Non-decomposing approaches

Geometric patterns can handle regular, simple, and one-UAV operation path searches. No environment decomposition is needed in such cases. Early samples of this approach were standardized in Mission Planner, the popular flight control software, to actively cover the region. In this pattern, movements are straight lines that turn around at the end of the cycle at a closed angle. In the last situation, the movement passes the external end of the region and the beam goes back to the origin. Back-and-forth and spiral patterns are the most common pattern used here^25^.

### Path search algorithms

Evolutional algorithms are the main candidates for effective path planning and finding possible solutions in a short time. Let’s discuss the existing research.

#### Artificial potential field

The artificial potential field algorithm was first introduced in 1986 by a researcher named Khatib. This algorithm is based on simple mathematical calculations, so it was initially used traditionally for obstacle detection and path planning in robots. Over time, this method became one of the most basic algorithms for solving path planning problems. Due to the simple implementation of this method and low computational requirements, local algorithms are usually based on APF^26^. The artificial potential field algorithm uses the magnetic force method in an unknown environment. In the magnetic force, the attractive force is used to reach the target point and the repulsive force is used to prevent collision with obstacles. This algorithm assigns an artificial potential field to each point in space using potential field functions. One challenge for the potential field algorithm is the local optimal trap. The local optimal trap occurs when all artificial forces (attractive and repulsive) neutralize each other. The local minimum condition means that if the UAVsis in that condition, it cannot continue its path^27^. Researchers have proposed two approaches to eliminate the local minimum problem in the artificial potential field method. In the first approach, when the local minimum problem occurs, the solution method changes to another solution method such as circumventing the obstacle boundary^28^. This auxiliary method can effectively solve the local minimum problems, but in this technique, the path length is not considered and we usually have a longer path than the best answer. The second approach to solve the local minimum problem is to use optimization methods to find the appropriate coefficients of absorption, repulsion and step length that can both pass the local minimum and also consider the path length in optimization^29^. These two proposed approaches to solve the local minimum problem can partially solve the APF method problem, but both have drawbacks. The obstacle boundary circumvention method to prevent the local minimum increases the path length. Optimization or combination approaches of APF with optimization algorithms can find a shorter path, but the convergence time of the local minimum is high. With the increasing complexity of the UAVswork environment, the requirements of path planning algorithms become more and more and the traditional path planning strategies cannot meet their needs. In order to adapt to the complex application environment and application requirements, there is an urgent need to design path planning algorithms with shorter paths and faster processes in complex environments. Among the path planning algorithms proposed by researchers, meta-heuristic algorithms have shown the characteristics of fast and accurate solution. Therefore, to solve the problems of heuristic algorithms, meta-heuristic algorithms have been studied that can solve the problem of getting stuck in the local optimum. These types of algorithms are not dependent on a specific problem and define a general strategy that is fixed for solving any problem. The only difference is that the problem of interest must be defined according to the existing strategy to be solvable. Genetic algorithm, ant colony algorithm, particle swarm optimization and butterfly optimization are all of this type that will be introduced and discussed in the following.

#### Ant colony optimization algorithm

This algorithm, a manifestation of collective intelligence, is inspired by the ant colonies. It has been used for path planning^30^. Authors in^31^ introduced a variant to create an environment model before path planning. They used a strategy to select the next path moves by the constructed model. It could save the path and includes information regarding the problem space^32^. The artificial ant colony algorithm adds new features such as local optimization, a priori knowledge, forecasting, and search methods to its natural peer.

*Particle Swarm Optimization (PSO)* is Another practical path-planning algorithm that does a global random population-based optimization^33^. In PSO, a group of particles move in a search space, each representing a candidate path. For each particle position, velocity, the best-experienced position, and the objective function corresponding to that best-experienced position properties are adjusted. Particles move in the search space to find the optimal path by updating each particle’s position according to the particle’s and the neighboring particles’ experience. Once a solution is found, a vector is drawn from the origin to the best-experienced position. The previous best position of the particle is recorded as the local path and the best position found by the swarm is called the best global path. PSO searches for an optimal solution by updating the position and velocity of each particle.

#### Butterfly optimization algorithm

Butterflies use their smell, sight, taste, touch, and hearing to find food. Their sense of smell is the strongest one. A butterfly generates a fragrance of specific intensity proportional to its fitting. This perfume is spread broadly and other butterflies can sense it. Once a butterfly smells the fragrance of another butterfly, it moves toward the source. This is called the global search. A butterfly smelling no fragrance moves randomly. This is called local search. In the butterfly optimization algorithm (BOA) each butterfly has its fitting which distinguishes the algorithm from other meta-heuristic algorithms^16^.

Intelligent optimization algorithms such as genetic (GA)^34–37^ and ant colony (ACA)^38,39^ are used in searching the path step. The former has suitable maneuverability in global searching. It can quickly find all the solutions without falling into the local optima^40^. The latter is better at finding solutions^37^. It solves the problem through empirical results. However, the common limitations of these algorithms are barely avoidable in large-scale UAV path planning. The genetic algorithm performs weak in low-performance breath first searches needing a large number of iterations and converges quickly^35^. The ant colony algorithm is sensitive to the initial parameter setting. An inappropriate setting reduces the amount of the search and gives poor results^37^. In $BOA$ each butterfly has its unique fragrance perceived through different sensory receptors^16^. This might be useful in UAV path planning considering the effective parameters. We propose a butterfly algorithm focusing on reducing the length and cost of the path and avoiding collision with the obstacles.

## The proposed method

Most path-planning algorithms are suitable for two-dimensional and robotic applications. They can not be applied to the three-dimensional space directly. We introduce a meta-heuristic UAV path planning algorithm in three-dimensional space that traverses a minimum length path, reduces energy consumption, and does not collide with obstacles. The UAV maneuvers are reduced using generated intermediate points. This reduces energy consumption and falls into the local optima. We then compare the performance of the proposed algorithm with common meta-heuristic ant colony and particle swarm optimization algorithms^29^. The path cost of those algorithms is compared with ours in two-dimensional and three-dimensional spaces against obstacles modeled as simple geometric shapes such as circles and cones. Our method adds an intelligent throwing agent to BOA. Its fitting function is based on the distance traveled, operational power of the UAV, and the number of collisions with the obstacles. By using the intelligent agent, we create intermediate points that reduce UAV maneuvers and energy consumption. A pseudo-code of the proposed algorithm including the definition of the fitting and collision avoidance function is given in Appendix A.

### Modeling the environment

**Figure 3:**
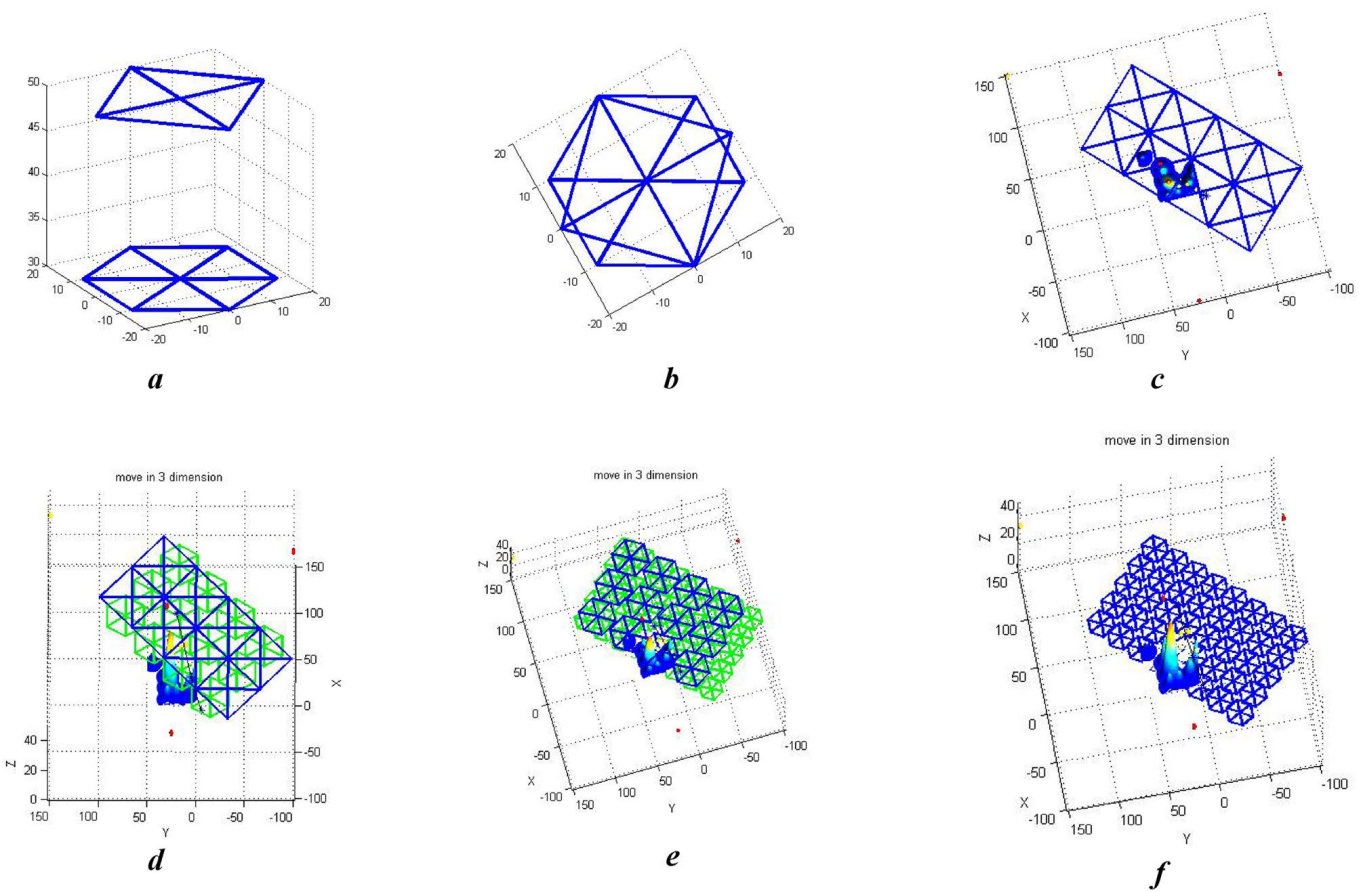
(**a**) The Decomposition of the environment using simple geometric shapes. (**b**) A quadrilateral container containing a hexagon. (**c**) A quadrilateral container containing a hexagon. (**d**) A quadrilateral container containing a hexagon. (**e**) A quadrilateral container containing a hexagon. (**f**) A quadrilateral container containing a hexagon.

In an application such as agriculture, construction or traffic monitoring, and fire management we need to cover the whole environment. The environment model is usually simplified in such cases by using simple geometric shapes such as convex or rectangular polygons or non-polygons such as circles and spirals^40^. The environment is searched using different algorithms according to the application. For instance, in non-polygons, morse functions are used to decompose the environment. They can handle the obstacles and use back-and-forth and spiral flight patterns in the search. The latter pattern moves along the main axis and turns 90 degrees in turning maneuvers. They need less computation time to find covering paths. However, they have limited durability since the vehicle should reduce its speed, turns, or accelerate during the turning maneuvers which increases the flight time, the impact of a collision with the obstacles, and subsequently the consumed energy. Also, the installed sensors on the UAV increase its weight and reduce its durability^41^. The cells decomposed as convex polygons use a network representation. Such a representation increases computation time but simplifies the implementation and provides more accurate and clear images^17^. Figure 3 shows different model griding methods and the containers’ capacity.

Large-scale flight environment simulation involves performance preservation challenges. It makes the simulation a significant part of the process. To improve the algorithm’s performance, the environment model should be simplified as much as possible without losing image clarity. The geometrical simplification is therefore controlled using an approximate error threshold. Different paths in the environment can be rendered in different levels of detail. This means preparing the image for depiction after modeling and simplifications to enhance the performance. Most existing preprocessing and modeling methods model environments in a planar simple way^42^. This simplification works for applications such as cleaning floors, detecting land mines, and lawn mowing. Our algorithm uses a multi-level altitude-based simplification. This reduces the random states in the path search step and adds to the computation speed. Multi-level environment modeling is performed after the simplification with or without decomposing. In the decomposing approach, the environment is divided into convex geometrical shapes. In non-decomposing approaches, the environment is searched dissecting it into smaller pieces. The decomposes approach is used more due to its convex cells that reduce the intensity of turning maneuvers and reduce UAV energy consumption.

### Modeling the environment cellular decomposition

Existing research mostly decomposes the environment into geometrical shapes using criteria such as the number of points in the environment. Using such criteria needs knowing the exact position of the points and lots of computations. Errors in input data affect the decomposition greatly and add significant overhead. We use the criteria of angle and altitude for a multi-level decomposition. These criteria require less computation than coordinate criteria and are used more in path planning. Equations (9)–(14) show the containers’ cellular decomposition.

9 $$x = sidelength \times \cos^{^\circ } \left( {angles} \right)$$

10 $$y = sidelength \times sin^{^\circ } \left( {angles} \right)$$

11 $$X = \mathop \sum \limits_{i = 1}^{n/m} x + sidelength \times d\left( i \right) \times m$$

12 $$Y = \mathop \sum \limits_{i = 1}^{n/m} y + sidelength \times d\left( i \right) \times m$$

13 $$Square = func\_G\left( {X,Y,Z - C} \right)$$

14 $$hexagonal = \mathop \sum \limits_{i = 0}^{n} \mathop \sum \limits_{j = 1}^{2n} func\_G\left( {X + \left( \frac{sidelength}{b} \right) \times d\left( j \right)} \right) + \left( {i \times \left( \frac{sidelength}{b} \right),Y + \left( \frac{sidelength}{b} \right) \times d\left( j \right) - \left( {i \times \left( \frac{sidelength}{b} \right),Z - C} \right)} \right)$$

In the first step, optimal values for parameters such as population size, maximum number of iterations, number of butterflies, and number of samples in each iteration are obtained using greedy methods. The random, uniformly distributed initial points are then distributed in the environment. Many intermediate points are generated using the agent and the UAV is guided to the new positions according to the path fitting and step parameters. The maximum length of each agent in the throwing function is $n$. The length of the throwing parameter is $n$ and plans the path globally. In early steps, an unfit path is penalized less due to the lower value of $Step$. As we get close to the final steps, we expect to select more appropriate paths as there are fewer points ahead and a wrong path selection receives heavier penalties. BOA determines the values of $i$ and ${p}_{i}$.

$$\underbrace {i}_{{{\text{First}}\;{\text{step}}}}\;\;\underbrace {{p_{1} \;\; \cdots \;\;p_{i} \cdots \;\;p_{2} \cdots p_{n - 1} \;\;p_{n} }}_{{{\text{Second}}\;{\text{step}}}}$$

In these equations $angles$ vector and $sidelength$ variable represent the angles and side length of the geometrical shapes. Vectors $X ,Y,x,y$ are the coordinates of the points calculated from the angle vectors and the side length variables. $Z$ Is the UAV’s altitude. $d$ and $k$ vectors determine whether the polygon moves up, down, left, or right. $m$ and $b$ parameters make the shapes bigger or smaller according to their level. $func\_G()$ is a function used to draw lines between points and $C$ is a constant parameter.

### Intelligent throwing agent

**Figure 4:**
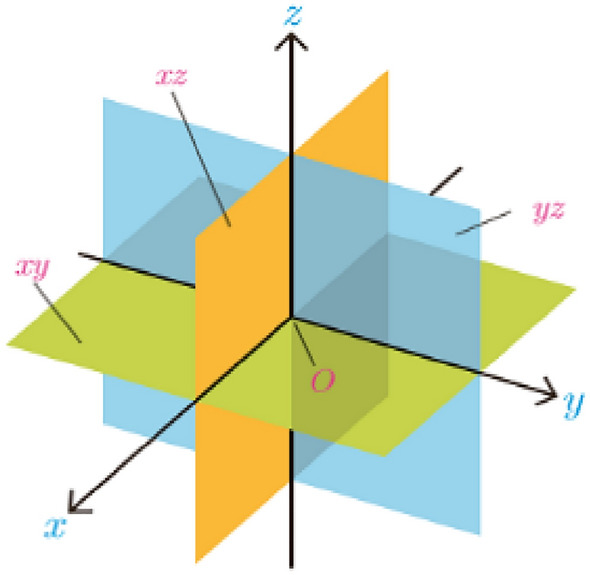
Intended regions for an intelligent throw.

Our algorithm is the first to use a butterfly optimization algorithm for path planning. It goes through global and local search steps. Choosing a globally optimal path and avoiding falling into local optima needs a supervisor who decides intelligently based on the path fitting. This intelligent agent deletes the wrong points and throws the UAV as necessary. This virtual agent assists the UAV in fitting and choosing the path. It avoids falling into local optima and increases the network coverage during path planning. Figure 4 show Intended regions for an intelligent throw.

The intelligent throw in the problem space is performed as follows:

- Determine the intermediate point for throwing
- Determine the length and direction of the throw (throwing size)
- Determine the intermediate point for the throw
- Determine the length and direction of the throw (throw size)
- Move from regions $1\mathrm{ to }8$ to the optimal solution point (Fig. 4).

Using an intelligent throwing agent during $BOA$’s path determination has two advantages. The points are generated randomly in a wide specific area of the environment and therefore don't get stuck in local optima. Also, due to the point’s variable length, the algorithm calculates the length and path of the throw for UAV transport in a wider space to avoid getting close to global optima.

### Fitting function

The algorithm consists of initialization, iterations, and final steps. The algorithm finishes if an optimal solution is found after initialization and iterative searching. The steps are:

- Define the objective function, solution space, and parameter values. Determine the initial number of butterflies (whose total number is constant).
- Define the butterflies’ positions, fragrance storage, and fitting
- Create artificial butterflies and begin to search.

Reducing path length and energy consumption, and avoiding UAV collision with the obstacles affect calculating fitness function. This section and the next one discuss the equations used for calculating the targets.

15 $$d\left(x,y\right)=\sqrt{{(x-y)}^{2}}$$

16 $$pl = \left\{ \begin{gathered} \mathop \sum \limits_{i = 1}^{L - 1} pl_{i - 1} + d\left( {p_{i} ,p_{i + 1} } \right),\quad if\;L > 0 \hfill \\ 0,\quad \quad \quad \quad \quad \quad \quad \quad \quad \quad \;o.w \hfill \\ \end{gathered} \right.$$

In Eq. (15) $d(x,y)$ is the Euclidian distance between the UAV’s current position and the intermediate point in the problem space in meters. In Eq. (16) $L$ is the number of intermediate points between the source and destination. We first calculate the distance between the source and the first intermediate point. The algorithm should calculate all the paths to the intermediate points, while there is an intermediate point, and make the best decision. The agent’s forward paths are examined and paths longer than the operational range of the UAV are penalized to become of lower priority. Assuming sufficient UAV power to cover the path, Eq. (17) chooses the intermediate points and new UAV position based on the distance and operational power of the UAV.

17 $$e = \left\{ \begin{gathered} \mathop \sum \limits_{i = 1}^{L - 1} e{ }_{i - 1} + 1 - \left( {d\left( {p_{i} ,p_{i + 1} } \right) - OP{ }_{i} } \right),\quad if\;d > OP \hfill \\ 1,\quad \quad \quad \quad \quad \quad \quad \quad \quad \quad \quad \quad \quad \quad \quad \;\;o.w \hfill \\ \end{gathered} \right.$$

$OP$ is the UAV’s operational power in terms of the distance it can transport in meters. If the distance from UAV to the next point is more than its operational power, a negative value is added to the path to penalize it. This makes paths longer than the UAV’s operational power unselectable. Once a new position is found according to the distance and operational power parameters, the path quality must be evaluated according to the number of obstacles in the paths and the collision impact criteria. We use two algorithms to calculate collision. In spherical obstacles, collision paths should be calculated to determine possible collisions. The quality of the path is measured according to that parameter. The algorithm uses Eq. (18) to find the length of the line segment between the current ${(v}_{1})$ and new $({v}_{2})$ positions and the center of the path obstacles $(pt)$ using the formula for the distance of a point from a line. A distance less than the obstacle’s radius means part of or the whole path collides with the obstacle This length will then be added to the path-calculated collision length and the number of collisions is increased by one. Otherwise, the length and number of collisions for this path will be zero. Function $cross$ calculates the external product between $a and b$ and ${d}_{ptl}(pt,{v}_{1},{v}_{2})$ is the distance of a point from a line segment which calculates the distance between the line segment between ${v}_{1},{v}_{2}$ and the center of the obstacles.

18 $$\begin{gathered} a = v_{1} - v_{2} \hfill \\ b = pt - v_{2} \hfill \\ d_{ptl} \left( {pt,a,b} \right) = \frac{{\left| {cross\left( {a,b,2} \right)} \right|}}{{\sqrt {(a^{2} + b^{2} } }} \hfill \\ \end{gathered}$$

19 $$Ci = \left\{ \begin{gathered} \mathop \sum \limits_{1}^{{L_{i} }} Ci_{i - 1} + 1 + d\left( {cnt_{i} ,d_{ptl\left( i \right)} } \right),\;\;f\;d_{ptl\left( i \right)} < o_{r} \hfill \\ 0,\quad \quad \quad \quad \quad \quad \quad \quad \quad \quad \quad \quad \quad \,o.w \hfill \\ \end{gathered} \right.$$

Equation (19) calculates the number and impact of the agent’s collisions with the obstacles.${o}_{r}$ Is the radius of the obstacle and $d\left({cnt}_{i},{d}_{ptl(i)}\right)$ is the Euclidian distance between ${d}_{ptl}$ and the center of the obstacle. If ${Dis}_{ptl}$ is more than the radius of the obstacle, part of or the whole path collides with the obstacle. After calculating collisions in aerial obstacles, let us discuss calculating them in terrain cone-shaped ones. We draw a line segment from the source to the first intermediate point in three-dimensional space using the line equation. That’s the first segment of the path. The existence of collision(s) and its length are checked and calculated for all points on this line segment. Assuming ${p}_{0}(x,y,z)$ and ${p}_{1}(x,y,z)$ are the source and the first intermediate point’s coordinate, function $vec({p}_{0},{p}_{1},t)$ calculates all the points between those points with a distance $t$ and returns their coordinates. $z$ And ${z}_{out}$ are UAV altitude in that intermediate point and the obstacle’s altitude. $f$ Indicates whether there will be a collision. The criterion for the existence of a collision is calculated based on the topographic modeling of Eq. (7). Equation (20) finds the path length in cone-shaped obstacles. According to it, if the UAV altitude in the first point is less than the obstacle’s altitude and $f$ is zero, there will be a collision in the beginning moment. The coordinates of this point will be recorded as the first collision point. All further points with a $f$ value equal to one and an altitude less than the obstacle’s altitude (falling within the obstacle) are added to the path length. This continues until the UAV altitude exceeds the obstacle’s altitude and that path segment becomes collision-free. That point’s coordinates will be the collision’s end coordinates. Equation (15) shows the total whole path length obtained.

20 $$Co = \left\{ \begin{gathered} \left( {\begin{array}{*{20}c} {\mathop \sum \limits_{1}^{L} Co_{i - 1} + d(Cs_{i} ,Ce_{i} )} \\ {Ce = \left[ {x,y,z} \right] } \\ {f = 0} \\ \end{array} } \right),\quad \quad \;z > z_{out} \;and\;f = 1 \hfill \\ \left( {\begin{array}{*{20}c} {\mathop \sum \limits_{1}^{L} Co_{i - 1} + d(Cs_{i} ,Ce_{i} ))} \\ {Ce = \left[ {x,y,z} \right]} \\ \end{array} } \right),\quad \quad z \le z_{out} \;and\;f = 1 \hfill \\ \left( {\begin{array}{*{20}c} {Cs = \left[ {x,y,z} \right] } \\ {f = 1} \\ \end{array} } \right),\quad \quad \quad \quad \quad \quad if\;z \le z_{out} \;and\;f = 0 \hfill \\ \end{gathered} \right.$$

21 $$Cl=Ci+ Co$$

Now that the path length, operational power, and collision length are calculated, it’s time to calculate the fitting function. The algorithm iteratively improves the obtained points to find a final optimal path. The chance of selecting the wrong path and hitting the obstacles in the early steps is more than in the last steps. These values are constant in our algorithm. The algorithm specifies the path by connecting the source to the intermediate and destination points. The number and positions of the intermediate points vary according to the number of path obstacles and the complexity of the problem space. Different functions here have different significances. Determining the exact optimal value of these coefficients are beyond the scope of this paper and will be addressed in future publications. Equation (21) calculates the fitting function.

### UAV movements

Butterflies use the following equation to create fragrances in their new positions.

22 $$fragrance={sensory }_{modality}{ \times (fitness(H))}^{power}$$

$fragrance$ is the intensity and power of the smelled fragrance, ${sensory }_{modality}$ is the smelling-based sensory method, $fitness(H)$ is the stimulus intensity function correlated with a coded objectivity function, and $power$ is the power absorbed in different levels according to the sensing method. The status means the raw input of the sensors when talking about sense. Measurement of the energy and its processing is the same as in similar methods. $power$ and ${sensory }_{modality}$ can be chosen in [0,1] for most cases. These two parameters control the behavior of the algorithm and are significant in determining the convergence rate. The UAV behavior depends on two crucial variables of $fitness(H)$ function’s rate of change and the changes in $fragrance$ equation. In BOA the specific fragrance generated by a butterfly is proportional to its fitting. The algorithm uses a movement to determine the next move. Each butterfly’s position vector is updated as follows

23 $${x}_{i}^{t+1}={x}_{i}^{t}+{fragrance}_{\begin{array}{c}i \\ \end{array}}^{t+1}$$

Global and local search are the two key steps of the algorithm. The global search uses the following equation to move toward the most suitable butterfly or solution ($${g}^{*})$$.

24 $${x}_{i}^{t+1}={x}_{i}^{t}+\left({r}^{2}\times {g}^{*}-{x}_{i}^{t}\right)\times {fragrance}_{i}$$

${x}_{i}^{t}$ is the solution vector ${x}_{i}$ for $i$th butterfly in the $t$th iteration. $g*$ Is the best solution within the current iteration. ${fragrance}_{i}$ is the fragrance of the *i*th butterfly and $r$ is a random number in [0,1]. Once the target regions are selected, the next generated move to determine the points of the path will be smaller and more cautious. This determines the next optimal point at any moment. In the following local search, butterflies evaluate and save their path according to the intermediate points determined by intelligent throw. The local search uses the following equation

25 $${x}_{i}^{t+1}={x}_{i}^{t}+\left({r}^{2}\times {x}_{j}^{t}-{x}_{k}^{l}\right)\times {fragrance}_{i}$$

$p$ and ${x}_{j}^{t}$ are the ${x}_{k}^{t}$th and $j$th butterfly in the solution space. Artificial butterflies are now generated and the introduced agent iteratively updates the intermediate points according to the intermediate point and operational power. It decides on the next new position in the solution space by evaluating the fitting function. This guides the butterfly to the destination through the optimal path. The length of the agent (number of intermediate points) varies during this step. It will be maximum in the beginning but will change in run time. Two approaches can be used to update the butterfly's positions: ‌the BOA approach or using an intelligent throw function to avoid falling into local optima. The optimal path will be selected by calculating the performance of each butterfly. Switch $p$ is used to move between global and local searches. The iterations go on until the stop criteria, the maximum number of iterations. is met. At the end of iterations, the algorithm provides the best solution. Figure 7 shows a flowchart of the proposed algorithm.

**Figure 5:**
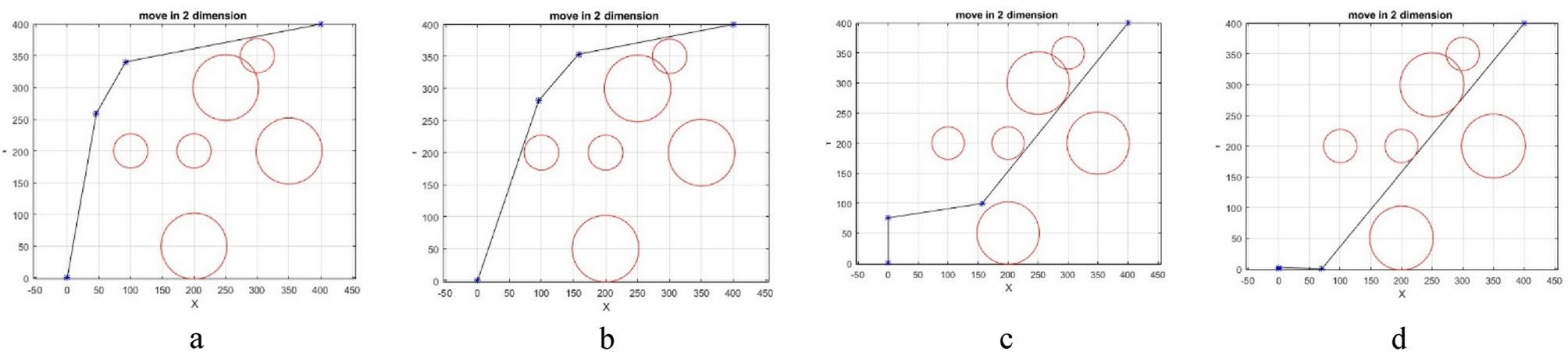
(**a**) Designing the initial path. (**b**) Improving the initial path. (**c**) Smart search and throw to another area. (**d**) Improving the final path.

In this section, the steps of path design are briefly shown in Fig. 5. It should be noted that in order to facilitate the transfer of concept, the UAVs movement is shown in two-dimensional space. It is obvious that in three-dimensional space, the algorithm’s performance will be similar. In this model, the position of obstacles and environment is fixed. The cells inside the circle indicate the obstacle and the cells outside the circle indicate the free area. The bottom left corner is the starting point and the top right corner is the target point. In this environmental model, the whole environment is first examined and based on the information of coordinates and obstacles in the environment, the initial path is designed (Fig. 5a). Such environmental modeling enables the UAV to have a basic understanding of global information and find an optimal path in the global environment^43^. In the next step, the previous path is optimized by repeating the algorithm (Fig. 5b). In the next steps, the length of the path determined is measured relative to the distance to the target point and if there is a shorter path in another area, the intelligent throw agent directs the algorithm to that area with an intelligent throw and draws a global path based on the new position. (Fig. 5c). Then the BOA algorithm improves the path designed in the new position in the next steps to achieve the shortest possible path (Fig. 5d). It should be mentioned that in some parts of the images where the path is tangent to the obstacle, a safe margin for the obstacles is considered, which prevents the UAV from colliding with the obstacle.

**Figure 6:**
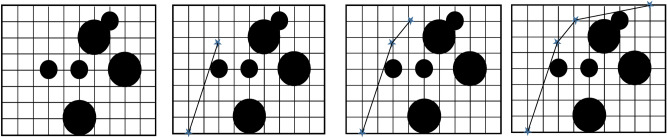
A screenshot of the second stage of route design.

**Figure 7:**
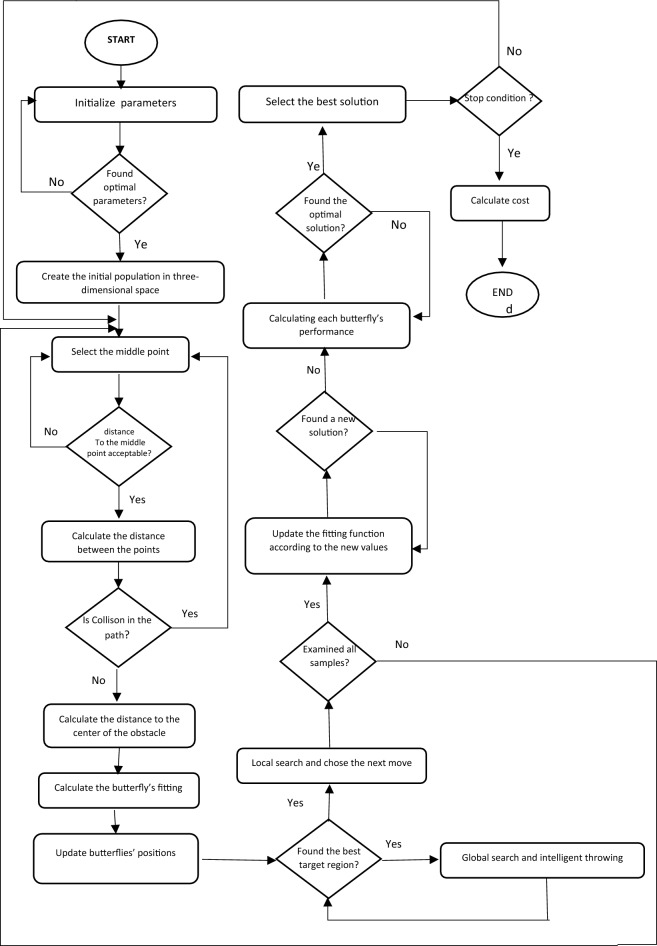
The Flowchart of the purposed algorithm.

The important point is that unlike greedy algorithms that select and draw a part of the path at each step, in the BOA algorithm, a global path is calculated at each step. To select a global path, intermediate points are used. Figure 6 is a screenshot of one of the steps of path design. The main goal of this algorithm is to design an optimal path from source to destination. Therefore, how to move on this path (moving straight, zigzag, etc.) is in the domain of motion planning problems, which will not be discussed in detail in this research (Fig. 7).

## Designing and evaluating the path in 2D space

Initial Parameters and defaults are set according to Table 1.

**Table 1:** Summary of the Parameters and simulation techniques.

	Parameters	Value
1	Simulation environment	Matlab
2	Population size	100
3	Beginning point	0, 0, 0
4	Endpoint	40, 40, 30
5	Test iterations	100
6	Number of butterflies	50
7	Simulation dimensions	2-D. 3D
8	Distance calculation strategy	Geometrical
9	Path selection strategy	Meta-heuristic
10	Input type	Optimal, random, constant
11	${w}_{2} ,{w}_{1}$	2 and 3, respectively
12	$OP$	3.5

**Figure 8:**
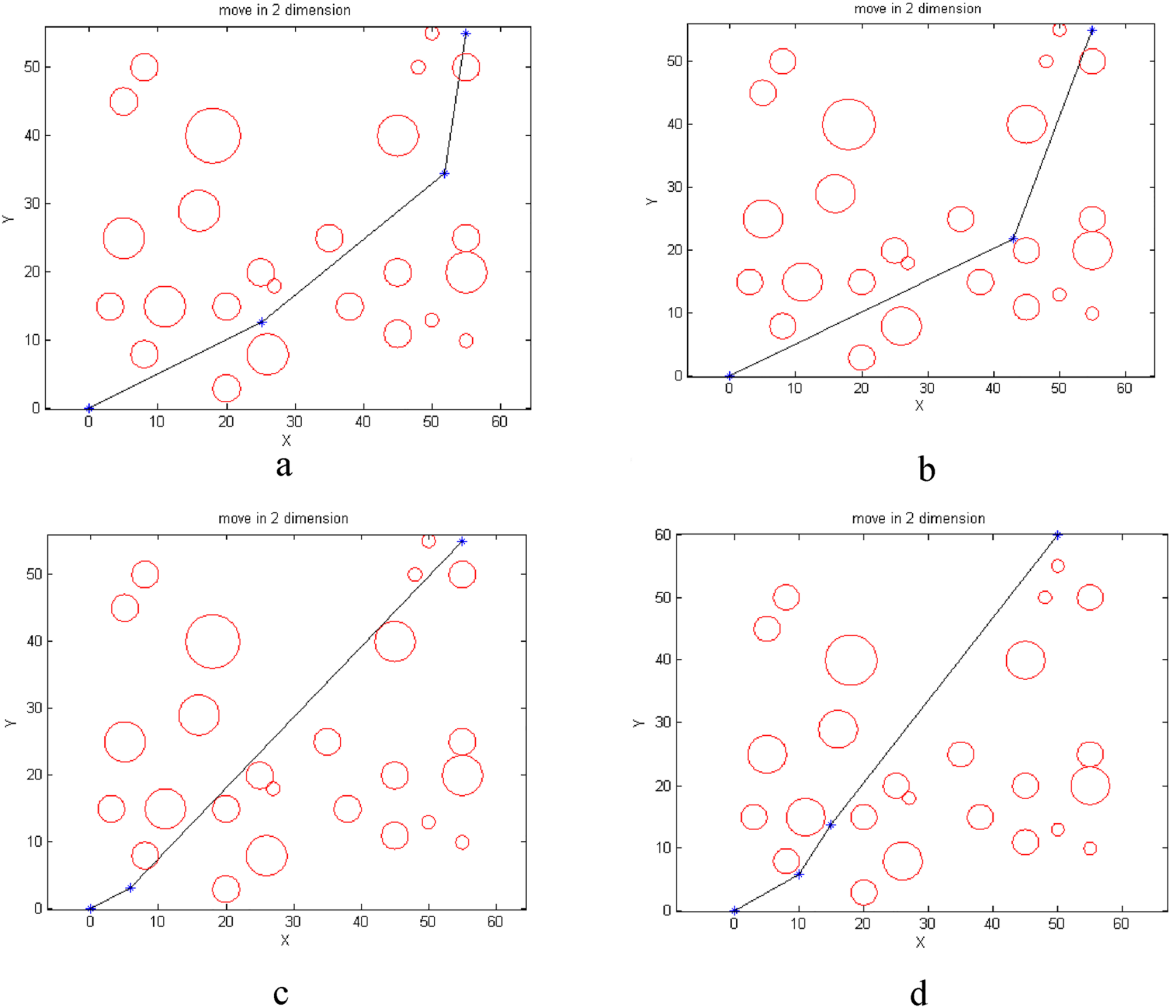
(**a**) Selecting the path with two intermediate points (step 1). (**b**) Selecting the path with two intermediate points (step 1). (**c**) Selecting the path with two intermediate points (step 1). (**d**) Selecting the path with two intermediate points (step 4).

The first test is performed on different spherical obstacles of varying radiuses located in specific positions. The algorithm finds an initial path based on two intermediate points which has a collision. This path is penalized to get a lower qualitative priority. However, since its early steps, it is accepted and the algorithm iterates. After some iteration, another path is found which contains one intermediate point and is tangent to the obstacle. While the number of collisions is still one, the collision length of the new path is shorter. The second path is deleted from the selection process and iterations continue. If the algorithm can find a shorter, better-quality path, the points are updated and the new path is drawn. Figure 8a indicates the path with two intermediate points. Figure 8b indicates the path with one intermediate point. Figure 8c indicates that after several iterations a path without collision or not tangent to an obstacle is found. Figure 8d shows selecting the path with less cost in the final step.

## Path planning in two-dimensional space with aerial obstacles-APF

**Figure 9:**
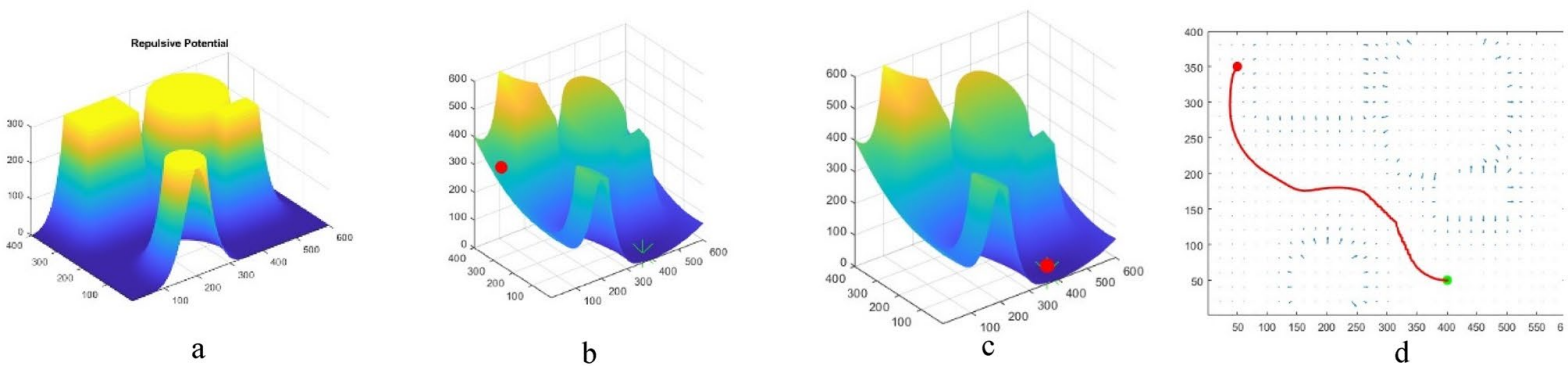
(**a**) Initial environment with 4 obstacles. (**b**) Starting the movement from the origin. (**c**) Following the path to the destination. (**d**) The path taken from the origin to the destination.

As shown in Fig. 9a, the initial environment with 2 circular obstacles and 2 rectangular obstacles has been simulated. The APF algorithm starts moving from the top left point and reaches the destination which is at the bottom right (Fig. 9b and c). In this scenario, the algorithm has been able to find a solution for the problem, but this solution is not necessarily optimal, because as shown in Fig. 9d, the path designed by the APF algorithm is curved. The reason for this situation is that this algorithm does not have a global view of the environment and in each step, it looks for a solution in the local area ahead. Therefore, if necessary, to avoid collision with obstacles or getting stuck in local optimum, it bypasses the obstacles. This causes the path length to be longer. While the proposed BOA algorithm, with the help of an intelligent throwing agent, can be guided to another area and, with a global view of the environment, choose the shortest path.

**Figure 10:**
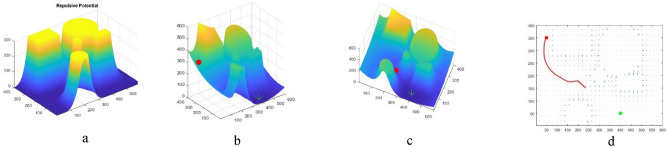
(**a**) Initial environment with adding an obstacle. (**b**) Starting the movement from the origin. (**c**) Getting stuck in local optimum. (**d**) Path from origin to stop at local.

In another scenario, while maintaining the previous conditions, a circular obstacle is added to the environment (Fig. 10a). As shown in Fig. 10b and c, the UAV moves from the starting point, top left, but due to getting stuck in the local optimum, the algorithm cannot find a solution and therefore the UAVs stops in the middle of the path (Fig. 10d). Therefore, it can be said that the APF algorithm shows a weak ability to overcome the trap of the local minimum by increasing the obstacles and provides suboptimal results. This is while the performance of the proposed BOA algorithm is optimal with increasing obstacles and even in crowded environments. Next, we will implement meta-heuristic algorithms.

From the simulation of the APF algorithm, we realized that this algorithm may have a reasonable time solution in some scenarios, but its weakness is getting stuck in the local optimum. Of course, different scenarios may have different results because the execution of the algorithm in real-world scenarios is difficult. From the results, it is clear that there is a trade-off between optimality and computational time constraints and the choice of algorithm should be based on both criteria and not necessarily one of them. Next, we will implement meta-heuristic algorithms.

## Path planning in three-dimensional space with aerial obstacles

**Figure 11:**
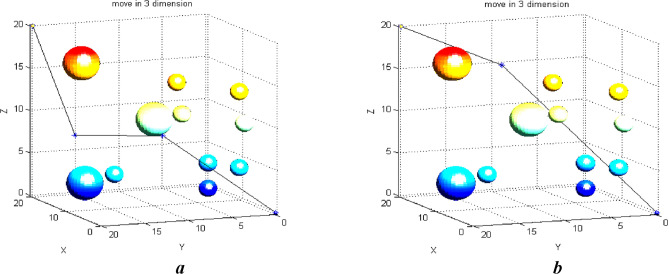
(**a**) Selecting the path using intermediate points and spherical obstacles. (**b**) Selecting the path using intermediate points and spherical obstacles.

In this section, we run the proposed algorithm using random parameters in a three-dimensional space that contains spherical obstacles. Figure 11 shows the algorithm's path selection steps. The UAV’s source and destination points are (0, 0, 0) and (20, 20, 20). The default population size is 50. number of butterflies in each population is 20, and The number of iterations is set at 300. The change of one coordinate during the generated path changes the path a lot. The path might change from one moment to the next. However, a better solution will be selected in the end. Parameters are chosen randomly at the beginning of this scenario.

## Path planning in a three-dimensional space containing terrain obstacles

**Figure 12:**
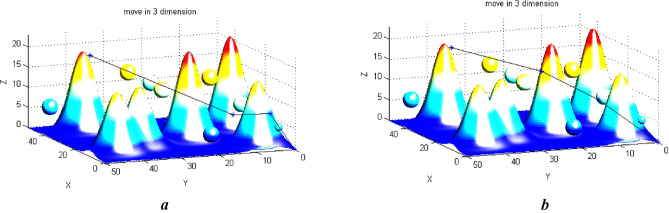
(**a**) Selecting a path using intermediate 2 points and combinational obstacles. (**b**) Selecting a path using intermediate 1 point and combinational obstacles.

UAVs are used as an effective hardware platform to monitor and petrol these complexes. There are difficult operational environments where humans or other devices can’t go. Path planning is necessary for applications such as search and rescue, traffic monitoring, and systematic patrolling in a wide geographical area with complex topographies. Many factors can affect the flight path. Due to the uncertainty of these operational situations, the optimal path must be optimal in all positions and not just between the two points. Let’s examine our algorithm’s performance in this situation. Figure 12 shows Selecting a path using intermediate points and combinational obstacles.

## Tests and results

**Figure 13:**
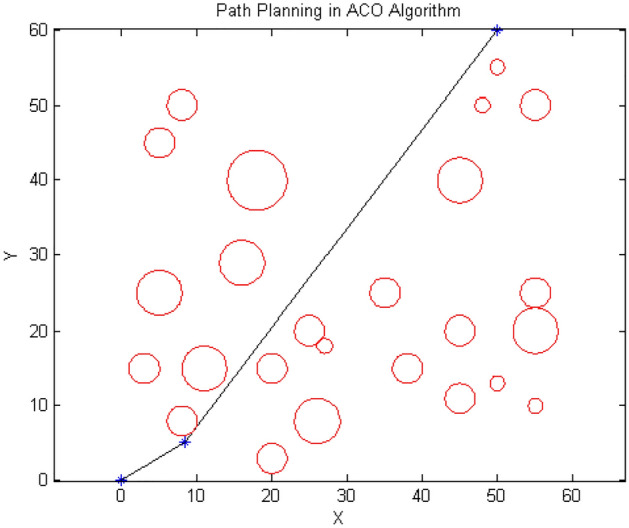
Path designed by ACO.

**Figure 14:**
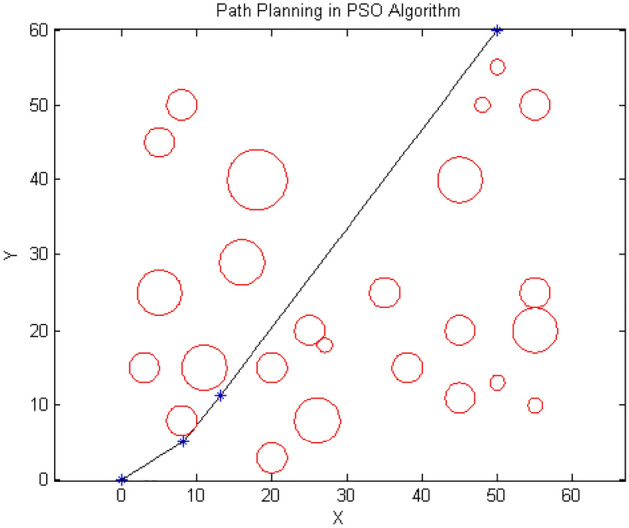
Path designed by PSO.

**Figure 15:**
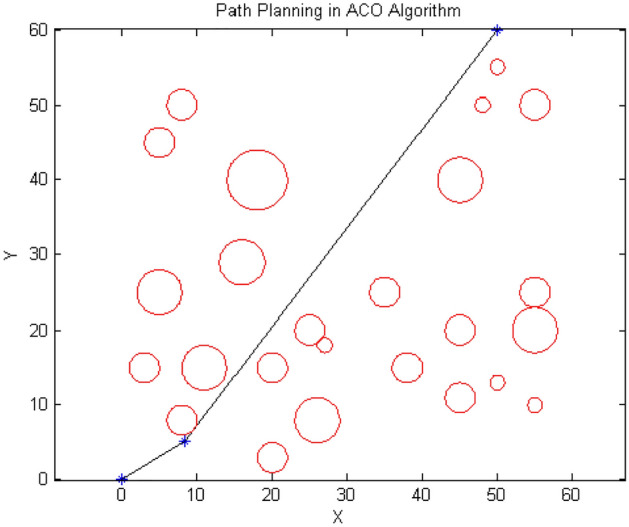
Path designed by ${\text{BOA}}$.

This section compares the results of running three algorithms in two- and three-dimensional spaces with random and optimal parameters according to their length and the cost of the path. Problem space is designed according to up-down cellular decomposition based on polygon shapes. The parameters are set the same as in the previous section. Figures 13, 14 and 15 demonstrate the paths designed by the algorithms in two-dimensional space using random parameters at the end of their runs.

**Figure 16:**
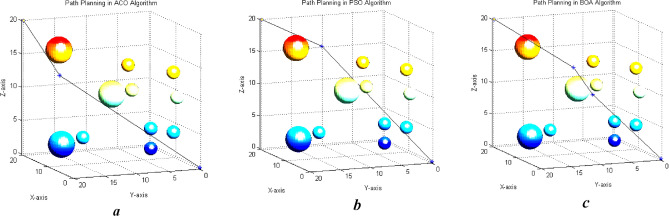
(**a**) Path designed by ACO. (**b**) Path designed by PSO. (**c**) Path designed by BOA.

**Figure 17:**
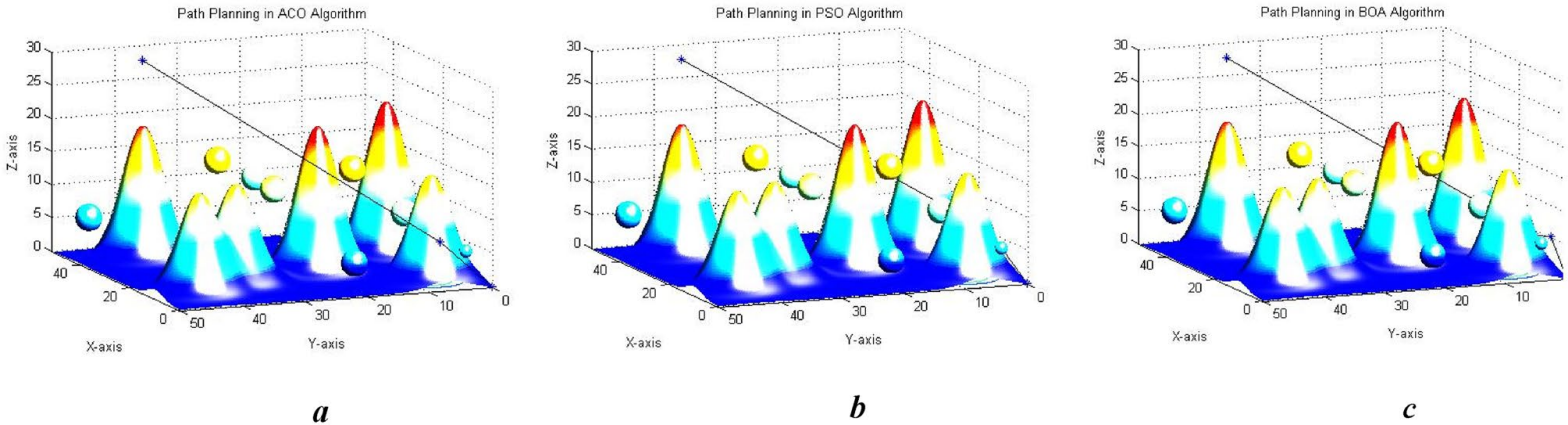
(**a**) Path designed by ACO. (**b**) Path designed by PSO. (**c**) Path designed by BOA.

**Figure 18:**
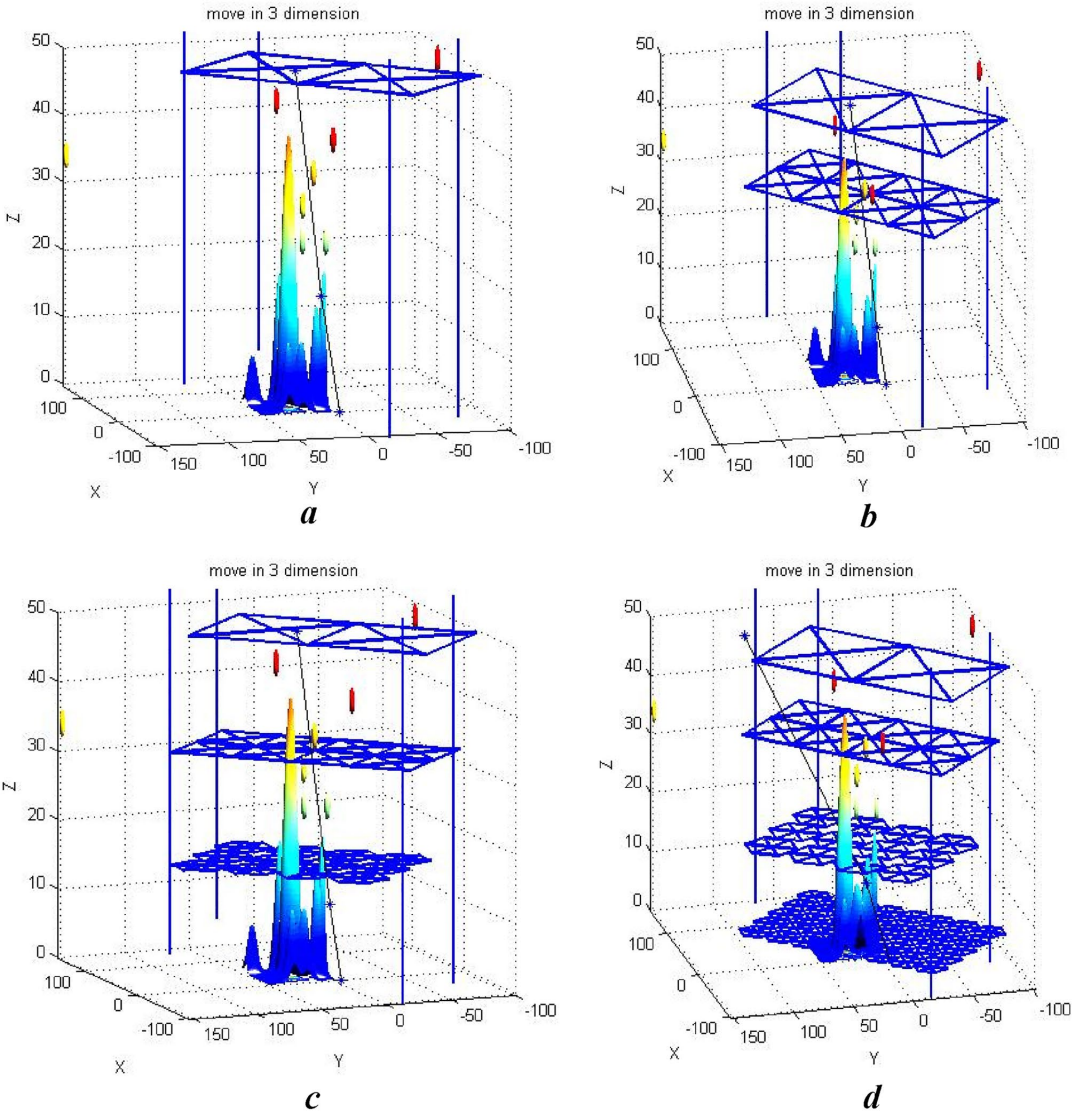
(**a**) Single-level decomposition. (**b**) Two-level decomposition. (**c**) Three-level decomposition. (**d**) Four-level decomposition.

According to Figs. 13, 14 and 15, the paths generated are fairly close to each other and only differ in the number and position of the intermediate point. For instance, ${\text{ACO}}$ uses one intermediate point, but ${\text{PSO}}$ and our algorithm use two points in different positions. Figures 16 and 17 show a path generated in three-dimensional space containing terrain and aerial obstacles. Figure 18 shows the single-level to four-level hierarchical cellular decomposition using quadrilaterals and hexagons.

**Figure 19:**
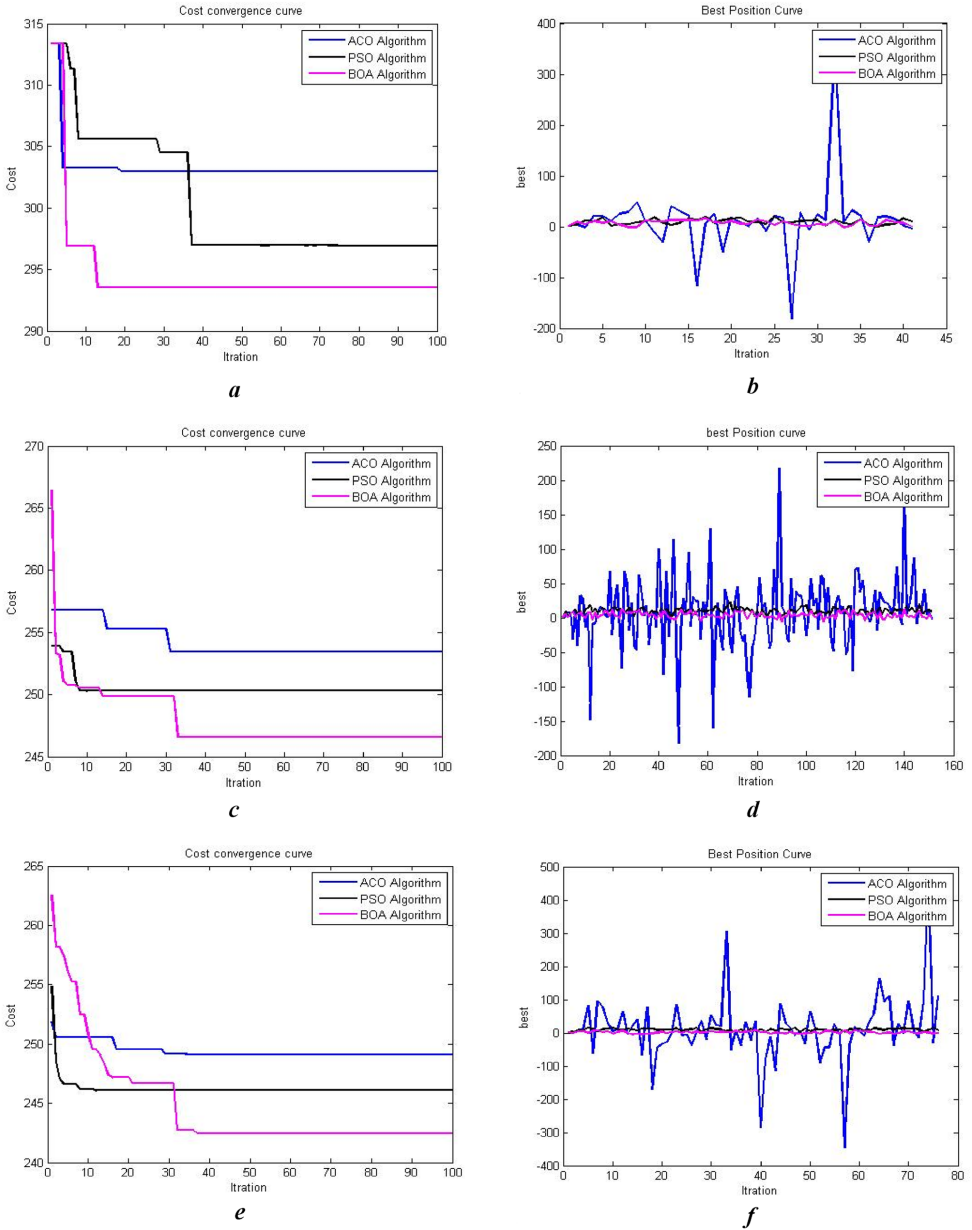
(**a**) Cost convergence curve in two-dimensional space using random parameters. (**b**) Cost convergence curve in two-dimensional space using random parameters. (**c**) Cost convergence curve in three-dimensional space using random parameters. (**d**) Best position curve in three-dimensional space using random parameters. (**e**) Cost convergence curve in three-dimensional space using optimal parameters. (**f**) Best position curve in three-dimensional space using optimal parameters.

**Figure 20:**
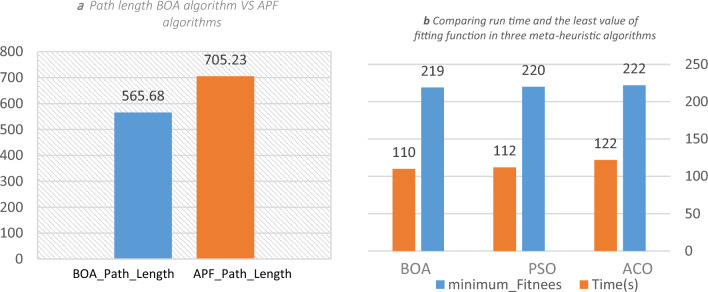
(**a**) Path length BOA algorithm VS APF algorithms. (**b**) Comparing run time and the least value of fitting function in three meta-heuristic algorithms.

Figure 18 shows the convergence curve for the ‘cost of path generation’ criteria and the best positions for ACO, PSO, and BOA according to their number of iterations in two- and three-dimensional spaces with aerial and terrain obstacles. The results indicate that the cost of $BOA$ with random parameters in two-dimensional space drops 20 units from 314 to 295 in 15 iterations. The cost remains unchanged after that. PSO cost decreases by 17 units from 314 to 297 in 40 iterations with no further changes in later iterations. ACO cost decreases 10 units from 314 to 304 in 10 iterations and remains constant afterwards. BOA achieves the least cost in the least number of iterations compared to the other two algorithms. The results of running algorithms in three-dimensional space using random parameters shows that BOA cost decreases by 20 unit from 265 to 246 in 32 iterations and remains unchanged afterwards. PSO cost drops 3 units from 254 to 251 after 10 iterations and ACO decreases 3 units from 257 to 254 after 30 iterations. BOA generally achieves the least cost through iterations while in other algorithms the cost doesn’t reduce with further iterations. Greedy algorithms are used to improve the results. The population-related parameters such as size and number are examined in all problem space states and their best values are chosen as the optimal input for the algorithm. Figure 17 shows the results of running the proposed algorithm with the optimal parameters. It shows that choosing optimal parameters using a greedy algorithm reduces the path costs in all three algorithms. The best position curve also has fewer fluctuations in different steps. Figure 19 indicates that BOA has a lower run time and fitting function values than ACO and PSO. We can conclude that BOA can replace much-used algorithms such as ant colony and particle swarm optimization in the path planning of airborne UAVs. Figure 20a indicates Path Length in APF and BOA. Figure 20b indicates Cost convergence curve and Best position curve in three-dimensional space using optimal parameters.

## Conclusions

Optimal path planning is a crucial problem in UAV pathfinding. This paper used a multi-level decomposition algorithm based on geometrical techniques. The proposed algorithm speeds up the computations by reducing the random states. We used meta-heuristic algorithms for path searching. These algorithms are practical solution-finding techniques due to their good ability to solve combinational multi-target problems. We used a butterfly optimization algorithm with an intelligent throw factor to improve the global search and avoid falling in local optima. The input parameters of the algorithm were calculated randomly and optimally. The results were compared to those of the ant colony and particle swarm algorithms. The cost convergence and optimal path indicate that the proposed algorithm has better performance than the mentioned algorithms under the same condition. It also had the least run time and fitting function value. Its planned path had better maneuverability against the obstacles which makes it acceptable in environments with many obstacles if the physical features of the UAV do not limit it. The algorithm was tested using optimal and random inputs. The results show that the performance of the algorithm with random inputs is close to optimal while it won’t suffer the optimal parameter computation overhead due to its multi-level modeling.

### Supplementary Information

Supplementary Information.

## Data Availability

Data will be made available on request. If someone wants to request the data from this study, contact the addres Hakimeh.mazaheri@gmail.com.
