# Supplementary material for: Path planning in three-dimensional space based on butterfly optimization algorithm
Source: Sci Rep. 2024 Jan 28;14:2332. doi: 10.1038/s41598-024-52750-9 (PMC11303821; doi:10.1038/s41598-024-52750-9)
Supplement: Supplementary file 1 — Supplementary Information. [file 41598_2024_52750_MOESM1_ESM.docx]

| **Appendix  A** | |
| --- | --- |
| $\mathbf{Algorithm1.} BOA-PATH\_PLANNING$ | $\mathbf{Algorithm2.}Fitness calculation$ |
| $1:Objective \boldsymbol{BOA\_function} f(x,y,z),x=(lenght,cost,collision),dim=no. of dimension$  $2:Generate initial population of n Butterflies b_{i}=(i=1,2,\ldots,n)$  $3:Stimulus Intensity Ii at xi is Determined by f(x_{i})$  $4:Define \mathrm{ensory}_{\mathrm{modality}}c, power exponent a and$  $probabibility switch P$  $5:\mathbf{While} stopping criteria not met \mathbf{do}$  $6: \mathbf{For each} butterfly in population \mathbf{do}$  $7:\mathbf{Call}Fitness calculation() using Algorithm2$  $8: Calculate fragrance for bf using Eq(9)$  $9: \mathbf{End} \mathbf{for}$  $10: Find the best bf$  $11: \mathbf{For each} butterfly in population \mathbf{do}$  $12: Generate a random number r from [0,1]$  $13: \mathbf{If} rand>p \mathbf{then}$  $14: Move towards best butterfly/ solution using Eq.(11)$  $15: \mathbf{else}$  $16: Move randomly using Eq.(12)$  $17: \mathbf{End} \mathbf{if}$  $18: \mathbf{End for}$  $19:Update the value of a$  $20:\mathbf{End while}$  $21:Output the best solution found$ | $1:Define uav velocity v, number of bf in each population NoG,$  $\mathrm{Population}$ $size NoI$  $2:\mathbf{While} stopping criteria not met \mathbf{do}$  $3: path length parameters generation$  $4: \mathbf{For each} butterfly in population \mathbf{do}$  $5:\mathbf{while} length is not zero \mathbf{do}$  $6: path length calculation$  $7: Remaining fuel\mathrm{calculation}$  $8: \mathbf{For each} \mathrm{Lenght}_{\mathrm{Pathpoint}}-1 not zero \mathbf{do}$  $9: \mathbf{For each} \mathrm{Lenght}_{\mathrm{Obs}}not zero \mathbf{do}$  10:$ptr calculation$  11: $cir\_collision\_lenght calculation$  $12: \mathbf{end for}$  $13:\mathbf{end while}$  $14:\mathbf{while} length is not zero \mathbf{do}$  $15: p_{i}=statr point$  $16: p_{1+1}=Mid point$  $17: \mathbf{For each} \mathrm{Lenght}_{\mathrm{Obs}}not zero \mathbf{do}$  $18$:$con\_collision\_lenght calculation$  19: $\mathbf{end}$  $20\mathbf{: end while}$  $21: fitness calculation$  $22: Collision lenght calculation$  $23: \mathbf{end} \mathbf{for}$  $24:\mathbf{end} \mathbf{while}$ |
| $\mathbf{Algorithm3.} Collision calculation$ | |
| $1:Objective \mathbf{function} \mathrm{mountains} obstacles z=\left( x,y \right)$  $2: \mathbf{For each}\mathrm{lenght}\mathbf{do}$  $3: normal distribution function \mathrm{calculation}$ $/ solution using Eq.(1)$  $4: \mathbf{end}$  $5:\mathbf{end}$ | |
| **Pseudocode of the proposed algorithm** | |
|  | |
| **Algorithm4.**  Find the optimal parameters | |
| $1:Define population size pop,number of bf in each$  $pop NoG, maximum number of generation M$  $2:Initialize the population random$  $3:\mathbf{For each} population size \mathbf{do}$  $4: \mathbf{For each}$number of gene in each chrom $\mathbf{do}$  5: $\mathbf{For each}$maximum number of generation $\mathbf{do}$  6: $Generate population$  $7: Generate Temp\_population$  $8: Update population$  $9:$ $\mathbf{Call} BOA\_function()$  $10: Calculate cost, \mathrm{best}_{\mathrm{pos}}, \mathrm{pop}_{\mathrm{size}},NoG,MG$  $11: \mathbf{end for}$  $12: \mathbf{end for}$  $13:\mathbf{end for}$ | |
| **Pseudo-code of the proposed algorithm in determining the initial population** | |
